# Predictors of Brain Natriuretic Peptide Serum Level Elevation in Patients with Symptomatic Chronic Subdural Hematoma: A Prospective Study

**DOI:** 10.3390/jcm10081791

**Published:** 2021-04-20

**Authors:** Mehdi Chihi, Ramazan Jabbarli, Ahmet Parlak, Marvin Darkwah-Oppong, Oliver Gembruch, Karsten Henning Wrede, Ulrich Sure, Homajoun Maslehaty

**Affiliations:** 1 Department of Neurosurgery and Spine Surgery, University Hospital Essen, University of Duisburg-Essen, 45147 Essen, Germany; ramazan.jabbarli@uk-essen.de (R.J.); ahmet.parlak@uk-essen.de (A.P.); marvin.darkwahoppong@uk-essen.de (M.D.-O.); oliver.gembruch@uk-essen.de (O.G.); karsten.wrede@uk-essen.de (K.H.W.); ulrich.sure@uk-essen.de (U.S.); 2 Department of Orthopedics and Trauma Surgery, St Vinzenz Hospital Dinslaken, University of Duisburg-Essen, 46537 Dinslaken, Germany; homajoun.maslehaty@st-vinzenz-hospital.de

**Keywords:** brain natriuretic peptide, chronic subdural hematoma, volumetry, trauma, biomarker

## Abstract

Background: Brain natriuretic peptide serum levels (BNP) on admission are frequently elevated in patients with symptomatic chronic subdural hematoma (cSDH) and predict unfavorable long-term functional outcomes. However, the reasons for these elevated levels remain unclear. Therefore, we aimed to identify the predictors of BNP elevation. Methods: Patients with unilateral symptomatic cSDH who were surgically treated in our department between November 2016 and May 2020 were enrolled. Patients’ symptoms and neurological deficits were prospectively assessed using a study questionnaire. On initial computer tomography, hematoma volumes and midline shift (MLS) values were measured to analyze the degree of brain compression. Results: In total, 100 patients were analyzed. Linear regression analysis showed that higher BNP levels were significantly associated with smaller hematoma volumes (*p* = 0.003) and littler MLS values (*p* = 0.022). Multivariate analysis revealed that presence of a neurological deficit (*p* = 0.041), a hematoma volume < 140 mL (*p* = 0.047), advanced age (*p* = 0.023), and head trauma within 24 h of admission (*p* = 0.001) were independent predictors of BNP elevation. Conclusion: In symptomatic cSDH, BNP elevation is related, among others, to the presence of neurological deficits and smaller hematoma volumes. Whether BNP elevation may coincide with the early stage of hematoma growth, i.e., immaturity of cSDH neomembrane, requires further investigations.

## 1. Introduction

Chronic subdural hematoma (cSDH) represents a common and frequent neurosurgical condition [1] with a growing incidence [2] and challenging outcomes after surgical management [3]. In contrast to the rapid expansion of an acute SDH, the irregular occurrence of microbleeds from the cSDH neomembrane generates inconsistent hematoma expansion, which often results in neurological deficits for cases of rapid expansion due to the brain being unable to adjust to this mass effect.

During the last two decades, several studies have shown that patient brain natriuretic peptide levels on admission (BNP) were elevated in many acute central nervous system (CNS) diseases, including stroke [4,5,6], hypertensive intracerebral hemorrhage (ICH) [7], aneurysmal subarachnoid hemorrhage (aSAH) [7,8,9,10,11] and traumatic brain injury (TBI) [12,13,14]. BNP has also been used as an indicator of disease severity [10,14] and as a predictor of long-term functional outcomes [12,15,16,17]. Although cSDH usually occurs at least three weeks after mild head trauma and does not represent an acute event relative to the aforementioned CNS diseases, we showed in a previous report that almost two-thirds of these patients unexpectedly presented with elevated BNP [18]. In a later report, we also demonstrated that BNP was a reliable predictor of postoperative long-term functional outcomes [19]. However, the reasons behind these increases in BNP levels remain unexplained.

Recent histopathological studies have demonstrated that immature cSDH neomembranes exhibit more bleeding and exudation and are associated with worse clinical presentations and the rapid expansions of small hematomas [20,21]. Based on these findings, we investigated possible predictors of elevated BNP in patients with symptomatic cSDH.

## 2. Materials and Methods

### 2.1. Study Population

**Figure 1:**
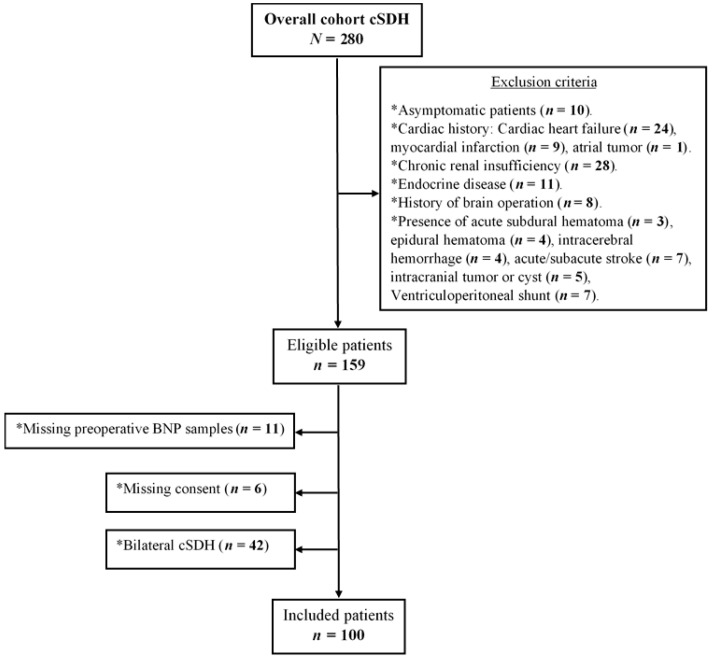
Study flow diagram.

All procedures were performed following the Declaration of Helsinki and the ethical standards of the institutional review board that approved this study (Medical Faculty, University of Duisburg-Essen, registration number: 15-6632-BO). Written informed consent was obtained from each patient or in cases of altered state of consciousness from the next of kin. We screened 280 patients with symptomatic cSDH who were surgically treated in our neurosurgical department between November 2016 and May 2020 (flow diagram in Figure 1). The term symptomatic was defined to include progressive headache, neurological impairments, seizures, and/or changes in behavior.

### 2.2. Inclusion Criteria

Patients ≥ 18 years diagnosed with a symptomatic cSDH and who underwent surgical removal of the hematoma were included. Hematomas had to be unilateral and isolated to allow for accurate assessment and analysis of brain compression.

### 2.3. Exclusion Criteria

Patients with cardiac or renal insufficiencies, a history of brain surgery, stroke, a ventriculoperitoneal shunt, intracranial tumors, or asymptomatic cSDH were excluded.

### 2.4. Treatment Protocol

The diagnosis of cSDH was made using cranial computed tomography (CCT, Somatom Definition AS, Siemens Healthcare GmbH, Erlangen, Germany; slice thickness = 5 mm) before surgery in all cases immediately after assessing BNP samples. Patients received a burr-hole trepanation and the insertion of a subdural drain for 48 h. Postoperatively, they remained in the intensive care unit (ICU) or intermediate care unit for 48 h. For these cases, a control CCT scan was performed on the second postoperative day, and the subdural drain was removed. However, some patients did not require any drainage or underwent operation through a minicraniotomy in case of septated cSDH or decompressive craniectomy in case of cSDH with acute bleeding and brain edema, without insertion of a subdural drain. For these cases, a CCT scan was performed on the next day. Perioperative antibiotic prophylaxis was ensured using a single injection of cefazolin (or erythromycin in case of penicillin allergy).

### 2.5. BNP Sample Collection and Management

Preoperative BNP levels were assessed on admission using an electrochemiluminescence immunoassay (Siemens, ADVIA Centauer^®^, Washington, DC, USA). The measuring range (provided by the manufacturer) was 2–5000 pg/mL.

### 2.6. Collection of Patient Data

Medical records were assessed by the first author (M.C.) using a study questionnaire on admission and included age, sex, comorbidities, such as atrial fibrillation, history of coronary heart disease, antiplatelet/anticoagulant therapy, and the presence of dementia. Patient symptoms and neurological deficits were assessed and patient neurological conditions using the Glasgow coma scale (GCS). Muscle force grading was done based on the medical research council manual muscle testing scale [22]. Hemiparesis was described as “severe” when the patient’s muscle strength was ≤ grade 3/5. Otherwise, hemiparesis was considered as “mild” (grade 4/5).

### 2.7. Radiological Measurements

Computer-assisted volumetric analysis was used on initial CCT to measure hematoma volumes. Hematoma margins were traced using iPlan software (BrainLab, Munich, Germany) for each axial CT-slice, and the volume was automatically calculated by the software in cubic centimeters (i.e., mL). Hematoma volume was assessed in all patients. Midline shift (MLS) was measured on initial CCT at the insular cortex’s level showing the basal ganglia and the thalamus above the basal cisterns. The midline was traced vertically from the frontal crest to the internal occipital crest. A perpendicular horizontal line was traced from the septum pellucidum to the midline. This distance was considered to be MLS and measured in millimeters. The presence of any hyperdensity areas on initial CCT, representing the microbleeds/hemorrhages of the outer membrane and/or septations of the hematoma, were documented. Volumetric analysis and Hounsfield units of the hyperdensity areas were measured using the iPlan software.

### 2.8. Statistical Analyses

All analyses were performed using IBM SPSS Statistics (version 25 for Windows, Chicago, IL, USA). Categorical data are presented as frequencies and percentages. Non-normally distributed continuous variables are expressed using the median and interquartile range (IQR). BNP values were log_10_-transformed (log_10_BNP) to assume a normal distribution when assessing linearity with hematoma volume values using a scatterplot. To assess statistical associations between log_10_BNP and hematoma volumes, a linear regression was performed. The Mann–Whitney U test was used to determine differences between two groups of a categorical variable on a non-normally distributed continuous variable (BNP). The factors associated with increased BNP using the univariate analysis that had *p-*values < 0.1 were then used in the multivariate analysis. A multiple regression analysis was then used to determine predictors for elevated BNP levels. *p*-values < 0.05 using two-sided testing were considered significant.

## 3. Results

### 3.1. Cohort Patient Characteristics and Operative Technique

In total, 100 patients (median age, 76 years, IQR 18 years; male/female, 3.8/1) were included in the study (flow diagram in Figure 1). Patients’ characteristics are summarized in Table 1.

### 3.2. Symptoms and Neurological Deficits on Admission

Patients’ consultations occurred due to symptoms and/or neurological deficits. The onset of nonspecific symptoms (headache, dizziness, change in behavior, cognitive deterioration, gait/balance disturbance) occurred relatively slowly and progressively over weeks/months. In contrast, seizures and neurological signs, such as alterations in the state of consciousness (GCS score < 15) and neurological deficits, such as aphasia, dysarthria, fine motor dysfunction and hemiparesis, resulted in more urgent consultations. The occurrence rates of symptoms and neurological deficits are summarized in Table 2.

### 3.3. Radiological Findings

**Figure 2:**
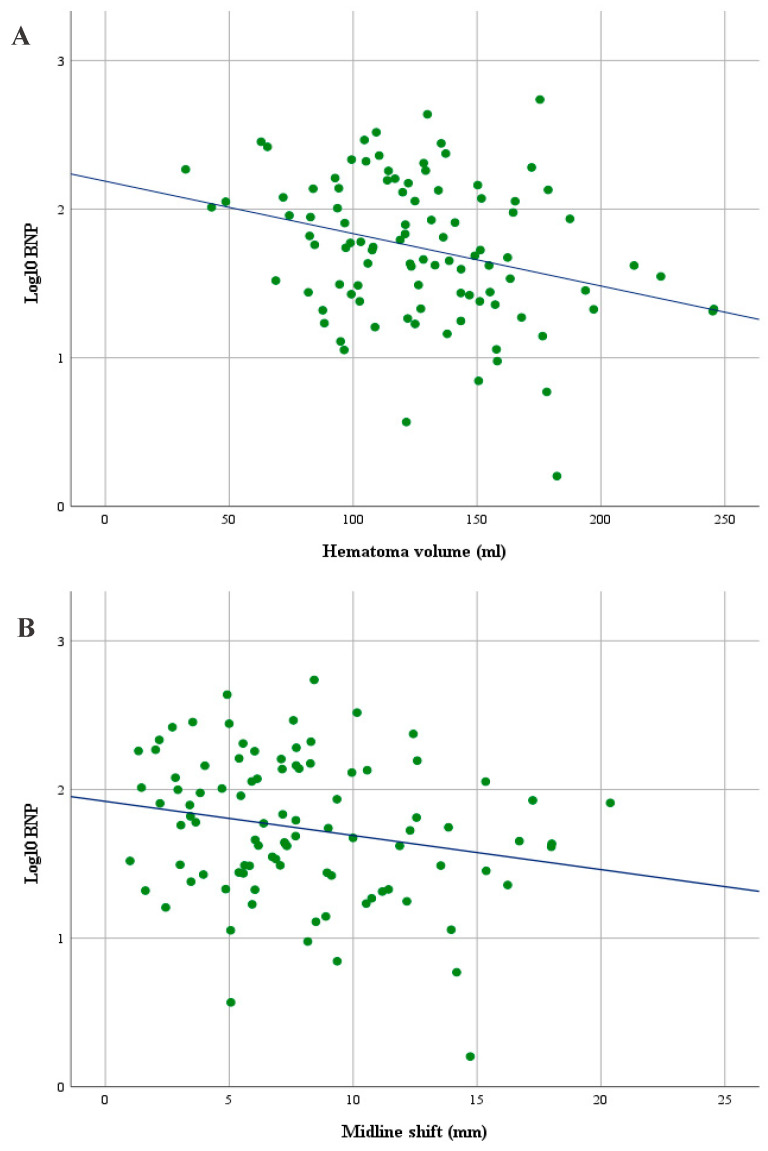
Scatterplot showing an inverse linear relationship between hematoma volumes and log_10_ brain natriuretic peptide serum levels (BNP) (**A**) and between midline shift (MLS) and log_10_BNP (**B**) in patients with symptomatic cSDH.

Linear regression was used to better understand the effect of hematoma volumes and MLS on BNP. To assess linearity, a scatterplot of log_10_BNP values against hematoma volumes and MLS values with a superimposed regression line was created. Visual inspection of these plots indicated an inverse linear relationship between hematoma volumes and log_10_BNP and between MLS and log_10_BNP (Figure 2). Higher log_10_BNP values were significantly associated with smaller hematoma volumes (*p* = 0.003) and littler MLS values (*p* = 0.022). The presence of hyperdensity areas, their volumes and mean Hounsfield units on initial CCT scans were not statistically associated with BNP values (Table 3). Furthermore, there was no statistical association between hematoma volumes and the presence of neurological deficits (Mann–Whitney U Test: hemiparesis, *p* = 0.264; mild hemiparesis, *p* = 0.283; severe hemiparesis, *p* = 0.756; dysarthria, *p* = 0.152).

### 3.4. Predictors of Elevated BNP Serum Levels

Elevated BNP values were significantly associated with advanced age (*p* = 0.0005), the presence of neurological deficits (hemiparesis and/or dysarthria) (*p* = 0.001), and head trauma within 24 h of admission (*p* = 0.024). In contrast, patients presenting with larger hematoma volumes, and greater MLS showed significantly lower BNP values (*p* = 0.006 and *p* = 0.022, respectively) (Table 3). As BNP was significantly associated with hematoma volumes, ROC curves analyses with different hematoma volume cutoff-values were performed. The ROC curve analysis with the best discrimination capacity was selected. The corresponding ROC curve analysis identified a hematoma volume cutoff-value of 140 mL and showed an acceptable discriminative capacity according to Hosmer et al. [23] with an area under the curve of 0.70 (95% CI: 0.58–0.80). After adjusting for all confounders, a multiple regression analysis revealed that the presence of a neurological deficit (*p* = 0.041), a hematoma volume < 140 mL (*p* = 0.047), advanced age (*p* = 0.023), and head trauma within 24 h of admission (*p* = 0.001) were independent predictors of increased BNP values. (Table 4). The multiple regression model statistically significantly predicted BNP levels, *F* (8, 91) = 5.482, *p* < 0.0005, *R*^2^ = 0.325.

## 4. Discussion

This study is the first to investigate the reasons behind BNP elevation in patients with symptomatic cSDH. We found that higher BNP levels are significantly associated with smaller hematoma volumes and littler MLS values. In addition, elevated BNP levels in cSDH could be, among others, independently predicted by the presence of a neurological deficit (hemiparesis and/or dysarthria) and smaller hematoma volumes.

The natural history of cSDH has been described as an initial occurrence of mild head trauma (mostly in elderly patients with brain atrophy) resulting in injury to the bridging veins of the dural border cells layer [24] and/or arachnoidea with subsequent leakage of blood and/or cerebrospinal fluid (CSF), respectively, in the new formed subdural space [25]. The spontaneous resolution of both fluid accumulations is common due to the highly active fibrinolytic systems contained in CSF [26]. However, a delay in absorption can occur due to advanced age or cortical atrophy, and the subsequent inflammatory and angiogenic responses of the body can lead to the formation of a neomembrane that encloses the hematoma [25]. The cSDH will continue to grow due to fluid exudation resulting from the hyperpermeability of the new fragile capillaries of the hematoma neomembrane and intermittent microhemorrhages of these blood vessels into the hematoma cavity [27]. The high concentration of tissue plasminogen activator within the neomembrane causes a local hyperfibrinolysis within the hematoma cavity and abnormal clotting of the hematoma [28].

The relationship between hematoma volumes and NT-proBNP (a non-active prohormone that is released from the same molecule that produces BNP) was first investigated in patients with hypertensive ICH in a prospective and multicenter study design, revealing significantly higher levels of NT-proBNP in patients with intracerebral hematoma volumes > 30 mL [29]. In addition, larger hematoma volumes were associated with increased ICP [29]. In patients with severe TBI [12,13,14], higher plasma levels of both NT-proBNP and BNP were also significantly associated with elevated ICP. Therefore, these data suggest that elevated BNP levels in acute CNS diseases are related to the increased ICP due to a higher degree of brain compression. Some authors suggested that the posttraumatic dysfunction of the autonomic nervous system, which leads to increased sympathetic activity and catecholamine hypersecretion, may represent the bridge between the brain and the heart [30], as an increase in catecholamine release could result in cardiac wall abnormalities and dysfunction [11,31] that may explain the BNP increase.

In contrast, in patients with cSDH, we found that higher BNP levels were, among others, independently predicted by the presence of neurological deficits and smaller hematoma volumes. These paradoxical results may be explained by the fact that ICP elevation in patients with cSDH remains an unpredictable event, which may occur many days/weeks after the initial trauma, but not in the few hours following admission, as in case of spontaneous ICH or severe TBI. In fact, due to the irregular occurrence of microbleedings and fluid exudation and inconsistency of their severity from one patient to another, some patients could experience “several microbleedings” of their small hematoma (originating from the outer membrane or hematoma septations) in a relatively short period. Consequently, even a small volume excess may lead to an elevation of the ICP and the occurrence of neurological deficits. Accordingly, Tomita et al. recently demonstrated that the assumption “thicker hematomas are associated with more severe motor weakness” in cSDH patients was logical but inaccurate. Indeed, the authors found that some patients with very thick cSDH had a minor motor weakness, suggesting that “hematoma thickness” was not a decisive factor in determining motor weakness severity [32]. These findings are in line with our results, as no statistical association was found between hematoma volumes and neurological deficits, in particular hemiparesis and its severity. Furthermore, the authors showed that “hematoma tension” on the motor cortex instead, calculated by Laplace law using hematoma thickness and hematoma pressure (intraoperative measurement using glass manometers), strongly correlated with the severity of the motor weakness [32].

The relationship between BNP levels and hematoma volumes in patients with cSDH has not yet been addressed. Recently published histopathological examinations [21] have revealed that immature cSDH membranes (type II neomembranes) that represent an early stage in the inflammatory cycle and exhibit more exudation and bleeding are associated with smaller, unstable hematomas and worse clinical presentations [20]. In contrast, patients with more mature hematoma neomembranes have already passed this phase and show larger and more stable hematomas, with a more gradual onset of symptoms [27]. Interestingly, our results showed that higher BNP levels in symptomatic cSDH, in contrast to acute CNS diseases, were significantly associated with smaller hematoma volumes and littler MLS values, suggesting that BNP elevation might coincide with the early stage of hematoma growth, i.e., the immaturity of cSDH neomembrane. This would then explains the inverse relationship between BNP values and hematoma volumes, as larger hematomas are more stable and evolve more slowly, allowing for adequate brain adaptation to the mass effect and a more gradual onset of symptoms [27]. Additionally, smaller hematoma volumes and the presence of neurological deficits on admission independently predicted higher BNP levels. These results also fit with our recent study findings regarding the prediction of poor functional long-term outcome through higher BNP levels on admission, which may promote using our recent predictive scoring system for cSDH long-term surgical outcome (the FLOP-score) [19] in the daily clinical practice.

### Study Limitations

Our findings do not represent the whole spectrum of patients with cSDH, only patients without premorbid conditions, such as chronic cardiac and renal insufficiencies and stroke. Further prospective studies with the larger patient–sample sizes and histopathological analyses of cSDH neomembranes must validate our findings.

## 5. Conclusions

Elevated BNP levels in cases of symptomatic cSDH are related, among others, to the presence of neurological deficits and smaller hematoma volumes, two arguments in favor of cSDH immaturity prompting hematoma expansion. Further histopathological studies must also consider analyzing BNP values on admission to investigate whether BNP can be used as a reliable predictor for neomembrane immaturity.

## Figures and Tables

**Table 1:** Patient characteristics and operative technique.

Comorbidities, Neurological Condition, and Operative Technique	*n*	%
Comorbidities		
Atrial fibrillation (AF)	11	11
Coronary heart disease (CHD)	11	11
AF and CHD	6	6
Antiplatelet/anticoagulant therapy	47	47
Dementia	11	11
Neurological condition		
GCS score = 15	53	53
GCS score < 15	47	47
Operative technique		
Burr hole trepanation	79	79
Minicraniotomy	19	19
Decompressive hemicraniectomy	2	2

**Table 2:** Symptoms and neurological deficits in a symptomatic chronic subdural hematoma (cSDH).

Parameters	*n*	%
Nonspecific symptoms		
Headache	24	24
Dizziness	12	12
Change in behavior	10	10
Cognitive deterioration	28	28
Gait and balance disturbances	38	38
Seizure < 24 h	9	9
Neurological Deficits		
Altered state of consciousness	32	32
Aphasia	31	31
anomic aphasia	22	22
expressive aphasia	7	7
receptive aphasia	1	1
global aphasia	1	1
Dysarthria	9	9
Fine motor dysfunction	16	16
Hemiparesis	27	27
mild hemiparesis	17	17
severe hemiparesis	10	10

**Table 3:** Relationship between BNP and clinical and radiographic parameters.

Parameters		BNP			
Mann–Whitney U Test	*N*	Medians (pg/mL)	U	z	*p*-Value
Headache	100	Yes (35.1)/No (73.4)	594.0	−2.567	0.010
Dizziness	100	*Yes (33.2)/No (56.7)*			0.088
Change in behavior	100	Yes (62.1)/No (51.2)			0.421
Cognitive deterioration	100	Yes (54.1)/No (56.7)			0.694
Gait and balance disturbance	100	Yes (44)/No (56.7)			0.276
Seizure < 24 h	100	Yes (103.0)/No (53.1)			0.309
Altered state of consciousness	100	*Yes (73.1)/No (45.5)*			0.063
Aphasia	100	Yes (64.8)/No (53.1)			0.554
Fine motor dysfunction	100	Yes (37.0)/No (56.7)			0.290
Gait ataxia	100	Yes (59.8)/No (54.1)			0.889
Dysarthria	100	Yes (95.0)/No (45.9)	585.0	2.114	0.035
Hemiparesis	100	Yes (112.3)/No (42)	1382.5	3082	0.002
Hemiparesis and/or dysarthria	100	Yes (93.1)/No (40.4)	1553.5	3440	0.001
Head trauma < 24 h	100	Yes (135)/No (48)	829.0	2.255	0.024
Presence of hyperdensity	100	Yes (53.1)/No (59.3)			0.942
Presence of acute blood	100	Yes (55)/No (57.6)			0.841
Hematoma volume (<140 mL)	100	Yes (73.4)/No (31.3)	692.5	−3.125	0.002
Spearman rank correlation		BNP			
Parameters	N	Correlation coefficient	*p*-value		
Age	100	0.413	0.0005		
Hematoma volume	100	−0.274	0.006		
Midline shift	100	−0.229	0.022		
Volume of hyperdensity areas	47	−0.083	0.584		
Mean HU of hyperdensity areas	47	0.122	0.420		

**Table 4:** Predictors of Increased BNP.

	BNP		
Predictors	B	95% CI	*p*-Value
Hemiparesis and/or dysarthria	37.067	1.553–72.581	0.041
Hematoma volume < 140 mL	−38.764	−77.062–−0.467	0.047
Age	1.829	0.257–3.400	0.023
Head trauma < 24 h	81.356	33.947–128.765	0.001
Headache	−16.177	−59.798–27.445	0.463
Midline shift	0.414	−4.848–5.677	0.876
Dizziness	−2.349	−54.087–49.390	0.928
Altered state of consciousness	37.154	−0.597–74.904	0.054

## Data Availability

The data presented in the study are available on request from the corresponding author. The data are not publicly available due to privacy restrictions.
